# Vangl as a Master Scaffold for Wnt/Planar Cell Polarity Signaling in Development and Disease

**DOI:** 10.3389/fcell.2022.887100

**Published:** 2022-05-11

**Authors:** Courtney A. Dreyer, Kacey VanderVorst, Kermit L. Carraway

**Affiliations:** Department of Biochemistry and Molecular Medicine and the UC Davis Comprehensive Cancer Center, UC Davis School of Medicine, Sacramento, CA, United States

**Keywords:** planar cell polarity (PCP), development, signal transduction, scaffolding protein, cancer biology

## Abstract

The establishment of polarity within tissues and dynamic cellular morphogenetic events are features common to both developing and adult tissues, and breakdown of these programs is associated with diverse human diseases. Wnt/Planar cell polarity (Wnt/PCP) signaling, a branch of non-canonical Wnt signaling, is critical to the establishment and maintenance of polarity in epithelial tissues as well as cell motility events critical to proper embryonic development. In epithelial tissues, Wnt/PCP-mediated planar polarity relies upon the asymmetric distribution of core proteins to establish polarity, but the requirement for this distribution in Wnt/PCP-mediated cell motility remains unclear. However, in both polarized tissues and migratory cells, the Wnt/PCP-specific transmembrane protein Vangl is required and appears to serve as a scaffold upon which the core pathway components as well as positive and negative regulators of Wnt/PCP signaling assemble. The current literature suggests that the multiple interaction domains of Vangl allow for the binding of diverse signaling partners for the establishment of context- and tissue-specific complexes. In this review we discuss the role of Vangl as a master scaffold for Wnt/PCP signaling in epithelial tissue polarity and cellular motility events in developing and adult tissues, and address how these programs are dysregulated in human disease.

## Introduction

Throughout embryonic development cells undergo extensive and dynamic expansion and rearrangements to form highly organized tissue structures. These processes are precisely spatiotemporally regulated and are largely dependent on the establishment and maintenance of polarity both within cells and across tissues. Epithelial cells are organized into polarized sheets that have two distinct yet overlapping polarity programs. These include apical-basal polarity, which ensures proper positioning of organelles and proteins along the apical-basolateral axis (Martin-Belmonte and Perez-Moreno, 2012; Rodriguez-Boulan and Macara, 2014), and planar polarity. Planar cell polarity (PCP) is the alignment of cells across an epithelial sheet orthogonal to the apical-basal axis. PCP is an evolutionarily conserved pathway that is mediated by a set of core components including, the seven-pass transmembrane receptor Frizzled (*Drosophila* Fz/mammalian Fzd), the atypical cadherin Flamingo (Fmi/Celsr), the tetraspanin-like transmembrane protein Van Gogh (Vang or Stbm/Vangl), and the cytosolic components Dishevelled (Dsh/Dvl), Diego (Dgo/Ankrd6), and Prickle (Pk) (Devenport, 2014). These core components are all required, and loss of a single core component disrupts PCP.

**FIGURE 1:**
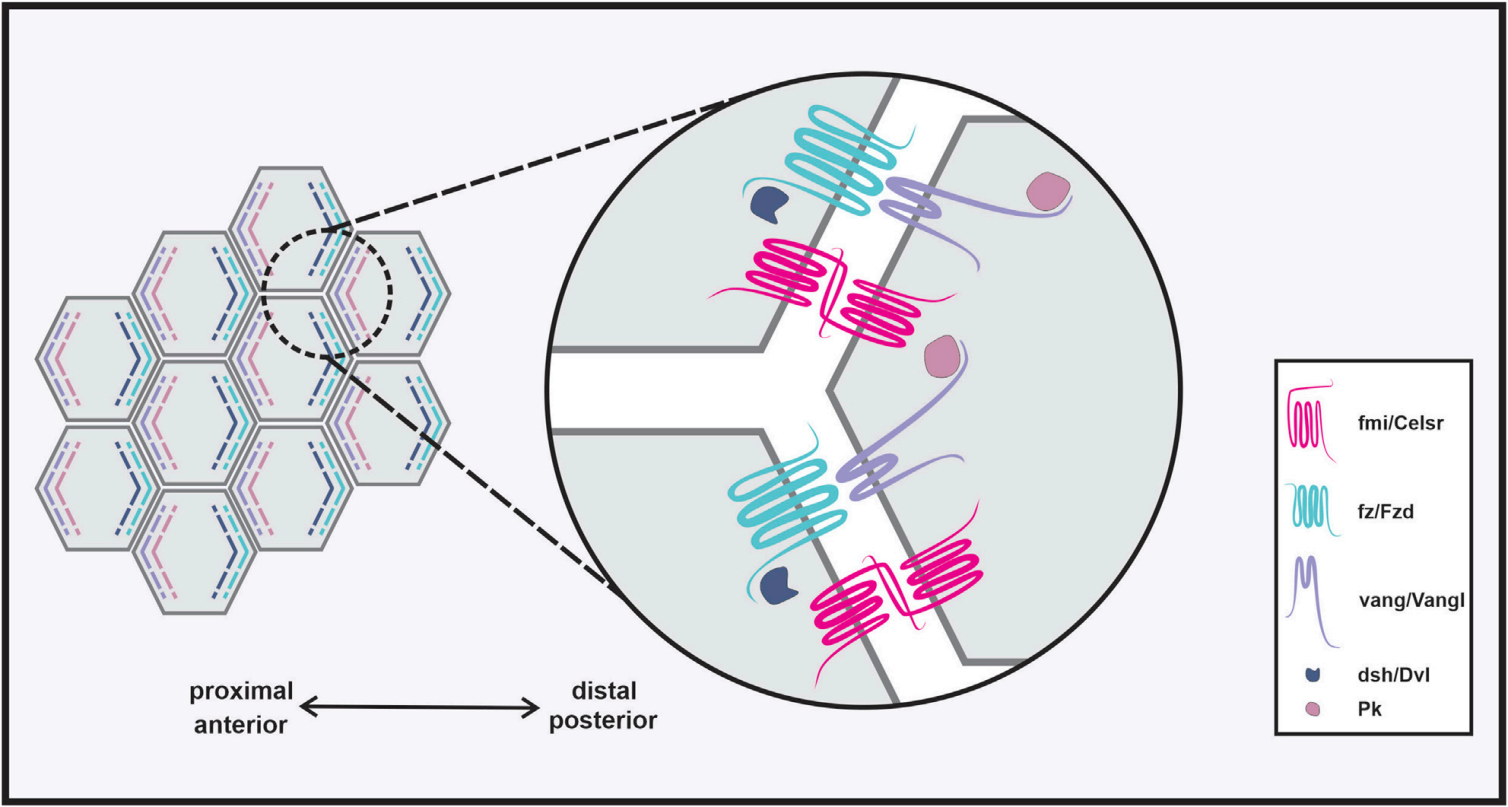
Establishment of planar cell polarity. Planar cell polarity (PCP) is the organization of cells across a tissue plane orthogonal to the apical-basal axis. PCP is established through asymmetric distribution of core component complexes, where Vangl-Pk and Fzd-Dvl complexes localize to opposite sides of the cell. Cell polarity is maintained and propagated to neighboring cells through intercellular protein-protein interactions between opposing complexes, and homophilic interactions between Fmi/Celsr.

PCP is critical for regulating epithelial tissue integrity and developmental processes by integrating global directional cues and producing local polarized cell behavior. Establishment of PCP within a tissue is regulated by several distinct mechanisms. PCP components are dynamically trafficked from the endoplasmic reticulum (ER) and then actively recruited to the apical membrane. There, components are organized into distinct complexes where Vangl-Pk and Fzd-Dvl are asymmetrically distributed to opposite sides of the cell [Figure 1; (Tree et al., 2002a)]. Asymmetric localization is maintained intracellularly by mutual antagonism between opposing complexes, and reinforced by intercellular protein-protein interactions between adjacent cells (Cho et al., 2015). Importantly, Wnts appear to provide instructional cues to PCP components that mediate component complex asymmetry and global polarity of epithelial tissues (Qian et al., 2007; Wu et al., 2013; Chu and Sokol, 2016), although this is still debated in the field (Ewen-Campen et al., 2020; Yu et al., 2020).

PCP also regulates cell migratory events critical for proper embryonic development. While some of the regulatory mechanisms are consistent with those within non-motile tissues, key differences exist. For example, in contrast to the establishment and maintenance of epithelial tissue polarity, PCP components largely rely on cues from Wnt ligands to regulate cell motility (Heisenberg et al., 2000). One important outstanding question is whether the classic asymmetric distribution of opposing complexes observed in epithelial tissues is recapitulated in migrating cells.

Scaffolding proteins such as Vangl critically regulate many key signaling pathways by mediating protein-protein interactions and organizing proteins into complexes. In polarized tissues and cells, scaffolding proteins are important for coordinating spatiotemporal assembly of signaling complexes through sequestration of their binding partners at specific subcellular locations within cells (Good et al., 2011; Garbett and Bretscher, 2014). In PCP-mediated processes Vangl can be found in distinct multiprotein complexes that can translate spatiotemporal signaling cues into coordinated cell behaviors. In this review, we will discuss the diverse binding partners of Vangl, summarize their functions in tissue polarity and morphogenesis, and highlight how dysregulation of Vangl can alter protein interactions and result in human disease. While we acknowledge the existence of extensive literature on PCP signaling in diverse organisms, we will be focusing on only a subset of publications that provide mechanistic insight into Vangl contribution to PCP in development and disease.

## Vangl Structure Promotes Assembly of Diverse Binding Partners

The transmembrane scaffold Vangl has distinct domains that allow for the assembly of diverse signaling complexes. Two mammalian orthologs of Vangl exist, Van Gogh-like protein 1 and Van Gogh-like protein 2 (Vangl1 and Vangl2). Vangl2 was the first PCP gene identified in mammals through studies using the *Looptail* mouse (*Vangl2* ^*Lp*^), which harbors missense mutations in the C-terminus of Vangl2 (Kibar et al., 2001; Murdoch et al., 2001; Montcouquiol et al., 2003). Human and mouse Vangl share 99% amino acid identity, and Vangl1 and Vangl2 share 70% sequence similarity, but appear to function the same biochemically and are redundantly required for proper development (Torban et al., 2008; Song et al., 2010). *Vangl1* and *Vangl2* genes encode transmembrane proteins that contain four transmembrane domains, two extracellular loops, and intracellular N- and C-terminal tails (Kibar et al., 2001; Murdoch et al., 2001). Vangl homo- and hetero-dimerizes at the plasma membrane, and one study demonstrated that these interactions may occur independently of the N- and C-terminal intracellular domains, suggesting oligomerizations could occur within the transmembrane domains or extracellular loops (Belotti et al., 2012), however, further investigation is required to identify the precise domains involved in Vangl oligomerization.

**FIGURE 2:**
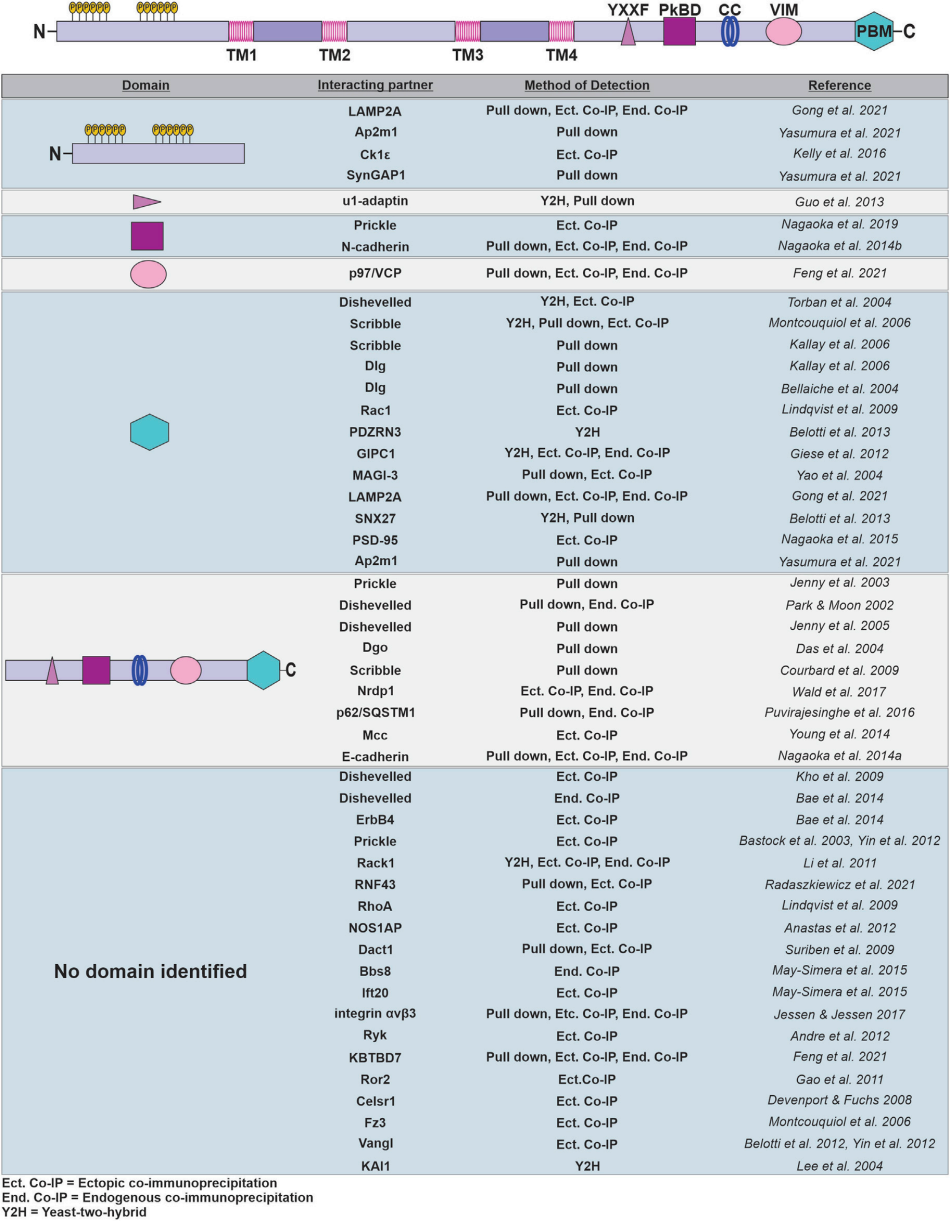
Structure and known binding partners of Vangl. Vangl is a four-pass transmembrane protein with intracellular N- and C-terminal tails. The N-terminus contains serine-rich regions that are phosphorylated upon Wnt stimulation. The large C-terminal tail contains several domains including a plasma membrane-targeting motif (YXXF), a Prickle binding domain (PkBD), a predicted coiled-coil region (CC), a VCP interacting motif (VIM), and a PDZ binding motif (PBM). Vangl has numerous known interacting partners, many of which have been mapped to its specific domains, or more generally to the N- or C-terminus. Binding interactions were assessed by pull down assays, yeast-two-hybrid screens, or ectopic or endogenous co-immunoprecipitations.

Many of the domains critical for the scaffolding properties of Vangl are located within the large cytoplasmic C-terminal tail. Both Vangls have conserved basolateral-sorting motifs (YXXF) critical for the recruitment of Vangl into transport vesicles, and proper cell membrane localization of Vangl (Guo et al., 2013; Iliescu and Gros, 2014). More recently, a p97/VCP-interacting motif (VIM motif) was identified in the C-terminal of Vangl2 that is required for direct interactions between Vangl2 and p97/VCP, a chaperone-related ATPase, which is critical for ubiquitination and degradation of Vangl through the endoplasmic reticulum-associated degradation (ERAD) pathway (Feng et al., 2021). The C-terminal tail of Vangl also contains a Prickle binding domain (PkBD) that facilitates interactions between Vangl and the cytosolic effector Pk (Nagaoka et al., 2019). Importantly, while Vangl lacks any known enzymatic activity, it contains domains critical for fostering diverse protein-protein interactions, including a PDZ binding motif (PBM) and a putative coiled-coil region in the C-terminus [Figure 2; (Kibar et al., 2001; Murdoch et al., 2001)]. Coiled-coil domains are structurally conserved across species and mediate various functions such as protein conformational changes and scaffolding molecular complexes (Truebestein and Leonard, 2016). However, the function of the coiled-coil domain in Vangl has been minimally explored and remains to be elucidated. The PDZ binding motif at the C-terminus of Vangl recruits other PDZ domain-containing cytoplasmic and membrane proteins and appears to be responsible for the recruitment and assembly of multiprotein complexes critical for PCP signaling. A diverse collection of proteins that interact with Vangl have been identified across multiple organisms and tissue types (Figure 2), reflecting the extensive impact of PCP signaling. While some studies identify the Vangl domains to which other proteins bind, many only broadly implicate the N- or C-terminus in binding, raising the possibility that there may be important domains or binding motifs of Vangl that have yet to be elucidated. Further studies are required to more precisely map these interactions to the exact domains or regions of Vangl, and to identify additional interacting proteins.

## Proper Localization of Vangl is Critical for Function

For Vangl to assemble its diverse interacting partners and function correctly, it must be properly embedded within a membrane (Iliescu et al., 2011). Mis-localization of Vangl results in impaired localization of other PCP pathway components as well as disruption of protein-protein interactions between Vangl and its partners. Mice with *Vangl2* ^*Lp*^ mutations display developmental defects as a result of impaired plasma membrane localization of Vangl2 (Iliescu et al., 2011). Notably, the *Vangl2* ^*Lp*^ mutation gives rise to more severe developmental defects than Vangl2 knockout in mice; this results from the entrapment of Vangl1 and other PCP proteins in the ER and loss of function at the cell surface (Yin et al., 2012).

As proper cell surface localization is critical for PCP component function, it comes as no surprise that protein trafficking plays a significant role in Vangl regulation. Newly synthesized proteins reach the plasma membrane by way of the ER-Golgi secretory pathway by way of delivery of packaged proteins in transport vesicles (Rothman and Orci, 1992). Mechanistic insight into Vangl2 trafficking to the plasma membrane emerged from the surprising observation that mice with a missense mutation in Sec24b, a component of the COPII vesicle protein complex that regulates ER-to-Golgi transport, displayed classic PCP defects (Wansleeben et al., 2010). Subsequent assessment of Sec24b loss of function mutations revealed that Vangl2 proteins were both mis-expressed and mis-localized (Merte et al., 2010; Wansleeben et al., 2010). *Vangl2* ^*Lp*^ mutant proteins fail to properly sort into COPII vesicles and remain trapped in the ER, suggesting that the *Lp* mutation prevents Sec24b from successfully sorting and trafficking Vangl2 to the plasma membrane (Merte et al., 2010). Others have demonstrated that export of Vangl2 from the trans-Golgi network requires Arfrp1, a GTP-binding protein, and the clathrin adapter complex AP-1. Here, Arfrp1 binds the μ1-adaptin subunit of AP-1 which binds to Vangl2 *via* the conserved YYXXF motif, suggesting that a Vangl2-AP-1-Arfrp1 complex transports Vangl2 to the plasma membrane (Guo et al., 2013). Interestingly, a distinct mechanism was identified for trafficking Fzd6 to the plasma membrane (Ma et al., 2018), suggesting that independent mechanisms exist for trafficking of different PCP proteins and likely contributes to the asymmetric localization of PCP components.

Importantly, many core PCP components, including Vangl, have been found to be post-translationally modified and this contributes to their localization and/or function (Gao et al., 2011; Kelly et al., 2016; Yang et al., 2017). Phosphorylation of Vangl is the most well-characterized post-translational modification and, in some cases, requires the activity of Casein kinase 1ε (CK1ε) (Gao et al., 2011; Kelly et al., 2016). Importantly, loss-of-function and hypomorphic mutations of CK1 result in classic PCP defects, thus identifying CK1 as an important component of the PCP pathway (Klein et al., 2006; Strutt et al., 2006). Later studies in the *Drosophila* ommatidia found that phosphorylation of Vangl occurs in a cell-autonomous manner through binding of CK1ε to the N-terminal tail of Vangl, and is required for the proper localization and asymmetric distribution of Vangl (Kelly et al., 2016). Similarly, the level of CK1ε-induced phosphorylation of Vangl dictates its stability and segregation into opposing complexes for functional PCP (Strutt et al., 2019).

Vangl phosphorylation also occurs in vertebrates and is important for tissue morphogenesis. In the developing limb bud, a Wnt5a gradient provides directional cues to determine the axis of elongation. Here Wnt5a induces the formation of a Vangl2-Ror2 complex, and Vangl2 is phosphorylated by CK1δ in a Wnt5a dose-dependent manner (Gao et al., 2011). Subsequent studies reported that Wnt5a-mediated Vangl2 phosphorylation is dependent on both CK1ε/δ and Dvl and is critical for asymmetric localization of Vangl2 and tissue morphogenesis in mice (Yang et al., 2017). Vangl2 is also reported to be phosphorylated by Fzd3 in *Xenopus* embryos*,* which impairs Vangl2-Pk3 binding, and may be critical to both mediating and maintaining asymmetrical localization of opposing PCP complexes (Chuykin et al., 2021). A recent study identified a novel post-translational mechanism for regulating Vangl protein levels. Feng et al. (2021) determined that Vangl2 proteins are basally phosphorylated in the ER prior to being transported to the cell membrane. Vangl2 proteins lacking basal phosphorylation are marked for endoplasmic reticulum-associated protein degradation (ERAD). Mechanistically, the ERAD-associated protein p97/VCP binds to the VIM domain in the Vangl2 C-terminus and recruits the E3 ubiquitin ligases CUL3 and KBTBD7 to mediate Vangl2 degradation *via* K48-linked polyubiquitination. Together, these studies highlight the diverse mechanisms that drive the proper localization of Vangl critical for its function as a scaffold.

## An Essential Role for Vangl in Establishing Planar Polarity in *Drosophila*

The *Drosophila* pupal wing provides a unique model for studying PCP as each wing cell produces a single hair at the distal edge that extends out distally, and proper hair positioning is dependent on PCP (Wong and Adler, 1993). Vang was originally identified as a polarity gene in *Drosophila* through a forward genetic screen to identify tissue polarity mutations (Taylor et al., 1998). The intracellular asymmetric distribution of opposing complexes that is required for the establishment of planar polarity was first observed and described in the *Drosophila* pupal wing and eye. Within a cell, Vang and Pk complex and localize proximally, while Fz and Dsh complex and localize distally [Figure 1; (Axelrod, 2001; Strutt, 2001; Tree et al., 2002b; Bastock et al., 2003)]. Vang-Pk and Fz-Dsh complexes are mutually antagonistic (Jenny et al., 2003) and this asymmetry is both established and maintained through modulation of PCP components themselves [reviewed in (Strutt and Strutt, 2009)].

Following the initial localization of PCP components to the apical cell surface *via* protein trafficking mechanisms, maintenance of apical localization of PCP complexes is primarily regulated through feedback interactions between the core components themselves (Das et al., 2004). From studies in *Drosophila*, it is apparent that Fmi is responsible for recruiting both Fz and Vang to the apical membrane (Chen et al., 2008). At the cell membrane, Fz recruits Dsh and Dgo to the apical surface, where they act in a feedback loop to maintain apical localization of other PCP components. Dgo physically interacts with the cytoplasmic tail of Vang and C-terminus of Pk and works redundantly with Pk and Vang to maintain apical localization of Fz and Fmi. Further, Dgo and Pk collectively promote and maintain apical localization of Dsh and Vang (Das et al., 2004). Others have demonstrated that the planar polarity established by asymmetric localization of complexes within a single cell is propagated to neighboring cells through the interaction of opposing complexes on adjacent cells (Chen et al., 2008; Wu and Mlodzik, 2008). Mechanistically, Vang physically interacts with Fz through the extracellular portions of both proteins which allows Vang to propagate asymmetry to neighboring cells and regulate non-autonomous signaling (Wu and Mlodzik, 2008). Together, these findings suggest that the formation of distinct multiprotein complexes is critical for intracellular interactions of PCP factors to maintain proper membrane localization.

Many of the regulatory mechanisms by which PCP factors establish and maintain opposing complex asymmetry and tissue-wide planar polarity are facilitated by the scaffolding characteristics of Vang that promote interplay between Vang and the cytosolic PCP factors. Most of these interactions appear to occur *via* the intracellular C-terminal tail of Vang. Vang binds both Pk and Dsh to recruit them to the cell membrane, and loss of Vang results in their mis-localization (Bastock et al., 2003). Conversely, Pk participates in the negative regulation of Vang at the cell membrane. Here, Vang recruitment of Pk results in Pk-mediated internalization of Vang-Fmi-Pk complexes through an endocytic pathway in the *Drosophila* wing (Cho et al., 2015). Vang scaffolding also allows Pk to differentially regulate Fz stability intracellularly and intercellularly. While Vang-Pk complexes stabilize Fz on adjacent cells, intracellular interactions between Pk and Fz-Dsh complexes destabilize Fz (Warrington et al., 2017). Further, Vang-Pk complexes negatively regulate Dsh-dependent activation of Fz signaling through the recruitment of Pk to compete with Dgo for Dsh binding (Jenny et al., 2005). In the *Drosophila* eye and wing, PCP establishment is at least partially mediated by physical interaction between Vang and Scrb, which occurs through the C-terminal tail of Vang (Courbard et al., 2009). These findings highlight the critical role of Vang as a scaffold upon which multiprotein complexes assemble to establish and reinforce planar polarity within *Drosophila* epithelial tissues.

## Vangl as a Regulator of Epithelial Polarity in Vertebrates and Mammals

Studies investigating PCP in vertebrate and mammalian tissues have demonstrated a conserved role for Vangl in establishing tissue polarity (Mitchell et al., 2009; Borovina et al., 2010; Guirao et al., 2010; Song et al., 2010; Vladar et al., 2012; Luo et al., 2021). While the precise mechanisms underlying Vangl contribution in these tissues are not well-defined, *in vitro* and *in vivo* studies point to a model for Vangl involvement. In Madin-Darby canine kidney (MDCK) cells, an epithelial cell line, Vangl2, membrane-associated guanylate cyclase with inverted orientation-3 (MAGI-3), and Fzd4 colocalize at cell-cell junctions. Vangl2 interacts with MAGI-3, and it appears this may recruit Fzd4 to a Vangl2-MAGI-3-Fzd4 complex to regulate downstream Rac/c-Jun N-terminal kinase (JNK) signaling and influence tissue polarity (Yao et al., 2004). Further, Vangl2 is critical in mediating proper MDCK cell polarization. Vangl2 interacts with polarity proteins Dlg and Scrib through the PBM in the C-terminal tail of Vangl2, suggesting that Vangl2 may serve as a scaffold for assembly of Vangl2-Dlg or Vangl-Scrib complexes to control cell polarity (Kallay et al., 2006).

The role of Vangl as a mediator of vertebrate planar cell polarity *in vivo* has been most extensively studied in the mouse cochlea. Similar to the *Drosophila* wing epithelium, the mouse cochlea consists of inner and outer hair cells which each contain a stereociliary bundle that projects from the apical surface. This results in an elaborate planar polarized pattern in the inner ear that allows for straightforward assessment of perturbations (Lu and Sipe, 2016). Vangl2 is required for the proper orientation of stereociliary bundles in the mouse cochlea, and *Vangl2* ^*Lp*^ mutations disrupt this. Interestingly, double mutant *Vangl2* ^*Lp/+*^ *;Scrb1* ^*Crc/+*^ mice displayed bundle orientation defects analogous to *Vangl2* ^*Lp/Lp*^ mice, indicating that the two may interact to promote proper stereociliary bundle orientation (Montcouquiol et al., 2003). Follow up studies (Montcouquiol et al., 2006) revealed that Celsr1-mediated recruitment of Vangl2 to the cell membrane promotes Scrb1-Vangl2 interactions *via* the C-terminal PDZ binding motif of Vangl2, and that this facilitates the proximal localization of Vangl2 within cochlear cells. Of interest, proper Fzd3 localization is Vangl2-dependent, and while it was initially reported that Vangl2 and Fzd3 both colocalize at the proximal edges of hair cells in the cochlea and physically interact, suggesting planar polarity in the mouse cochlea may not recapitulate classic PCP asymmetry observed in *Drosophila* (Montcouquiol et al., 2006), subsequent studies, using a higher resolution microscope, observed Vangl2 enrichment on adjacent hair cells, more indicative of classic PCP characteristics (Giese et al., 2012). Studies of the mouse cochlea have also identified novel proteins important for Vangl localization to the membrane. GAIP C-terminus interacting protein-1 (GIPC-1) interacts with Vangl2 at the C-terminal PBM and is critical for asymmetrical enrichment of Vangl during cochlear development (Giese et al., 2012). Similarly, Bbs8 and Ift20 coimmunoprecipitate with Vangl2 and may be critical in mediating the asymmetric accumulation of Vangl2 during the establishment of planar polarity in the cochlea (May-Simera et al., 2015). Most recently, Tower-Gilchrist and colleagues observed that the heterotetrameric protein complex AP-3 biochemically interacts with Vangl2, and loss of AP-3 impairs Vangl2 membrane localization, indicating a role for AP-3 in trafficking Vangl2 in the mouse inner ear (Tower-Gilchrist et al., 2019).

In other tissues, the mechanisms by which Vangl regulates tissue polarity are not as well defined but Vangl binding partners have been elucidated. In the developing mouse epidermis, Vangl2 is asymmetrically localized and physically interacts with Celsr1 to establish proper orientation of hair follicles (Devenport and Fuchs, 2008). In the female reproductive tract, mutations in Vangl2 cause defects in epithelial tissue organization and loss of actin cytoskeleton polarity due to mis-localization of Scrb1. These findings suggest that mutations in Vangl2 prevent it from serving as a scaffold for Scrb1, which may be critical for restricting Scrb1 localization (vandenBerg and Sassoon, 2009). Similarly, in the developing mouse pancreas, restriction of Vangl to the apical membrane is required for epithelial tissue integrity. Mis-localization of Vangl2 to basolateral membranes reduces Dvl2 abundance at the cell membrane and Rho kinase (ROCK) activity, resulting in epithelial tissue disorganization and pancreatic hypoplasia (Flasse et al., 2020). While there are some overlapping roles for Vangl in the establishment and maintenance of epithelial polarity in vertebrates to that in *Drosophila*, the requirement of PCP complex asymmetry and specific binding partners of Vangl have been more difficult to determine, highlighting the need for further studies in this area.

## Delineating Roles for Vangl in Mediating Cell Migratory Processes Critical for Proper Development

### Vangl Regulates Convergent Extension Movements Critical for Embryonic Development

Beyond regulating planar polarity in non-motile tissues, Vangl mediates dynamic morphogenetic events critical to proper embryonic development. The role of Vangl in convergent extension (CE), or tissue elongation along one axis with concurrent shortening of the other, is the most well-studied morphogenetic event to date (Murdoch et al., 2001; Jessen et al., 2002; Antic et al., 2010). Loss of Vangl results in a variety of dynamic developmental defects, most notably, neural tube defects (NTDs). NTDs have been most extensively studied in the *Vangl2* ^*Lp*^ mouse. In biochemical studies, both Vangl1 and Vangl2 bind all three members of the Dvl family. However, *Lp* mutations in Vangl fail to interact with any of the Dvl isoforms, indicating that this interaction, or failed Vangl localization to the plasma membrane, may be important in regulating CE during development (Torban et al., 2004).

Many studies in *Xenopus* have aimed to investigate the subcellular localization of Vangl and determine if classic PCP asymmetry is required for CE movements. In the *Xenopus* neural plate epithelium, ectopically expressed Vangl2 and Pk2 are asymmetrically enriched together at cell-cell junctions during cell intercalation, and this enrichment is tightly correlated with dynamic cell movements (Butler and Wallingford, 2018). Studies focused on uncovering the molecular mechanisms underlying Vangl contribution to CE movements in *Xenopus* and zebrafish have identified novel interacting partners for Vangl. A yeast-two-hybrid (Y2H) screen revealed that sorting nexin 27 (SNX27), a nexin protein important in endosomal recycling of transmembrane receptors, binds the Vangl2 C-terminal PBM, and this interaction is critical for mediating neural tube closure in *Xenopus* (Belotti et al., 2013). During zebrafish gastrulation, interaction of Rack1 and Vangl2 mediates Vangl2 localization to the plasma membrane (Li et al., 2011). During CE in *Xenopus* and zebrafish, Vangl2 interacts with Dvl through its C-terminal tail, and engages downstream signaling components JNK and AP-1, although it is not clear whether Vangl2-Dvl interaction is required for mediating these processes (Park and Moon, 2002).

Vangl contributes to actin cytoskeletal rearrangements, cell adhesion, and extracellular matrix (ECM) remodeling to regulate CE movements. During zebrafish gastrulation, Vangl2 regulates matrix metalloproteinase (MMP) localization and activity, which is critical for cell motility and ECM remodeling, although the precise mechanisms by which Vangl2 contributes to MMP trafficking remain to be defined (Williams et al., 2012a; Williams et al., 2012b). Vangl2 also engages motility pathways to mediate CE. For example, Vangl2 mediates cell rearrangements *via* RhoA and ROCK to regulate mouse neural tube closure (Ybot-Gonzalez et al., 2007). A later study determined that Vangl2 binds both Rac1 and RhoA. Here, Vangl2 and Rac1 interact through the Vangl2 C-terminal PBM to recruit Rac1 to adherens junctions and regulate cell adhesion and cytoskeletal rearrangements critical to mouse neural tube closure (Lindqvist et al., 2010). Vangl2-Dact1 interactions at the primitive streak are also critical for mouse germ-layer morphogenesis (Suriben et al., 2009).

Importantly, Wnts play critical roles in providing instructive cues to Vangl during gastrulation (Rauch et al., 1997; Heisenberg et al., 2000; Tada and Smith, 2000; Andre et al., 2015). Several Wnt ligands promote the formation of asymmetric Vangl2-Pk3 complexes, possibly through phosphorylation of Vangl2, and may ultimately regulate morphogenetic events critical to *Xenopus* development (Ossipova et al., 2015; Chu and Sokol, 2016). Vangl2 also mediates Wnt-dependent signaling through RhoA and JNK by interacting with Mutated in Colorectal Cancer (Mcc), a presumed tumor suppressor, through its C-terminus, to regulate CE in zebrafish (Young et al., 2014). Wnt ligands have also been reported to modulate Vangl protein levels during embryonic development. Andre et al. (2012) describe a mechanism in mammalian development where a Wnt-Ryk signaling axis stabilizes Vangl2 *via* interaction of Ryk and Vangl2. Vangl also contributes to cell migration in other developmental processes such as branching morphogenesis (Yates et al., 2010a; Yates et al., 2010b; Nagaoka et al., 2014a; Smith et al., 2019), but little mechanistic insight has been elucidated. Taken together, these studies highlight a functional role for Vangl in mediating CE in vertebrate development. In these contexts, Wnt ligands seem to provide the directional cues to Vangl, and Vangl engages multiple binding partners to precipitate downstream signaling events essential for proper CE in embryonic development.

NTDs in humans can also result from mutations in human Vangl1 and Vangl2. Several missense mutations in both Vangls have been identified in patients with NTDs, and these mutations were found to either diminish or completely abrogate Vangl-Dvl interactions (Kibar et al., 2007; Lei et al., 2010). Additional studies will be necessary to investigate the mechanistic outcomes of disrupted Dvl binding, but mutations in Vangl may prevent proper membrane localization of Dvl and promote Dvl degradation *via* the proteasome, ultimately resulting in NTDs.

### Vangl Regulates Cellular Movements Critical to Neuronal Development and Function

Early studies employing *Vangl2* ^*Lp*^ mice identified structural deformities in the developing brain (van Abeelen and Raven, 1968), pointing to a role for Vangl in brain development. Vangl is implicated in multiple processes required for proper neural development including neuronal migration, dendritic branching, axonal growth and guidance, and synaptogenesis, which are critical for the formation of functional neural networks (Martinez and Sprecher, 2020). Neurons require the appropriate spatiotemporal patterning to establish functional neuronal circuits, and this is largely achieved through directed cell migration (Ayala et al., 2007). Vangl2 mediates neuronal migration in multiple organisms (Bingham et al., 2002; Jessen et al., 2002; Glasco et al., 2012; Sittaramane et al., 2013; Davey et al., 2016), likely requires its C-terminus to assemble binding partners (Pan et al., 2014), and mediates signaling through engagement of downstream effectors JNK and ROCK (Vivancos et al., 2009).

Another critical feature of neurons is the extension of axons towards distinct targets. At the tip of axons are growth cones, which are highly motile structures that interpret signals in the extracellular environment to determine the direction of growth and guide the directed extension of axons (Chilton, 2006). Observations that *Vangl2* ^*Lp*^ mice have disrupted retinal axonal trajectories first implicated Vangl2 involvement in axonal guidance (Rachel et al., 2000). Subsequent studies supported these findings (Leung et al., 2016) and revealed that Vangl2 also mediates guidance of commissural axons, but a precise mechanism for Vangl was not reported (Fenstermaker et al., 2010; Onishi et al., 2013; Sun et al., 2016; Carvalho et al., 2020). However, mechanistically, Shafer et al. (2011) suggest that Vangl2 competes with Fzd3 for Dvl1 binding at the tip of filopodia in the growth cone, which enables the internalization Fzd3 receptors and proper steering of growth cones, driving axonal growth and guidance.

Developing neurons also undergo a rapid expansion of dendritic branches which are responsible for receiving inputs from neighboring cells. The size, shape, and location of dendrites are critical for their function, the inputs that they receive, and their ability to process neural information (Lefebvre et al., 2015). Loss of Vangl2, either by *Looptail* mutations (*Vangl2* ^*Lp*^) or null mutations in Vangl2 (*Vangl2* ^*-/-*^) significantly reduces the density of dendritic branches in the adult mouse forebrain (Okerlund et al., 2016). While the mechanistic underpinnings of these observations were not reported, a recent study found that Adaptor related protein complex 2 subunit μ1 (Ap2m1), a part of the heterotetrameric coat assembly protein complex 2 (AP-2), binds Vangl2 in the N-terminal intracellular tail to regulate dendritic branching (Yasumura et al., 2021). Together, these studies support a role for Vangl as a scaffold upon which mediators and regulators of dendritic branching may assemble, although the precise mechanisms by which Vangl governs dendritic branching during brain development requires further investigation.

Upon completion of sufficient dendritic branching, dendrites begin to form synapses between neurons. Synapses contain a structure called the postsynaptic density (PSD), which is critical for the assembly of receptors, ion channels, and other signaling molecules to produce neural networks (Waites et al., 2005). Intriguingly, Vangl2 binds to N-Cadherin, which interacts with β-catenin to regulate dendritic spine morphogenesis, in the PSD fraction of mouse brain extracts. The N-Cadherin-Vangl2 interaction occurs through the PkBD in the C-terminal tail of Vangl2 and the serine-rich β-catenin binding region of N-Cadherin, and this interaction is critical for Vangl2-driven Rab5-mediated endocytosis of N-Cadherin. The N-Cadherin-Vangl2 interaction is negatively regulated by both Pk and β-catenin which compete for binding of their respective partners to maintain appropriate levels of N-Cadherin, which is critical for normal synaptogenesis (Nagaoka et al., 2014b). Subsequent studies demonstrated that Vangl2 binds to PSD-95, a scaffolding protein that regulates signaling complexes in the PSD, through its C-terminal intracellular PBM, which enhanced binding between PSD-95 and Pk2, suggesting that Vangl2 plays critical roles in assembling multiprotein signaling molecules critical to synaptogenesis (Nagaoka et al., 2015). Together, these studies demonstrate a role for Vangl in neuronal development and suggest that Vangl may scaffold additional regulators of these processes that remain to be identified.

### Vangl in Cell Division

Oriented cell divisions play fundamental roles in cell-fate specification as well as tissue homeostasis. During development, cell-fate diversity is achieved through asymmetric cell division (ACD), a process where a cell divides to produce distinct daughter cells with unequal partitioning of cell-fate determinants that align with the orientation of the mitotic spindle. In the developing peripheral nervous system of *Drosophila*, a sensory organ precursor (SOP) cell generates four distinct progeny *via* a series of stereotyped asymmetric divisions and PCP signaling is necessary to orient this planar polarized cell division (Bellaïche et al., 2001a). In a SOP, classic PCP complex asymmetry, with Fzd-Dvl localized to the posterior cortex and Vangl-Pk localized to the anterior cortex (Bellaïche et al., 2004), initiates planar polarization of apical-basal polarity complexes, which are normally uniformly distributed around the cell cortex. In this context, Partner of Inscuteable (Pins) and Discs-large (Dlg), two members of the Dlg-Pins-Gαi apical-basal polarity complex that regulates the localization of cell-fate determinants, colocalize anteriorly with Vangl (Bellaïche et al., 2001a; Bellaïche et al., 2001b; Bellaïche et al., 2004). The anterior recruitment of Pins is Vangl-dependent and Vangl interacts with Dlg *via* its *C*-terminal PDZ binding motif, suggesting that Vangl both recruits and complexes with Dlg-Pins-Gαi to regulate cell-fate determinant localization and proper orientation of the mitotic spindle during mitosis (Bellaïche et al., 2004; Gomes et al., 2009).

Vangl appears to mediate ACD and cell-fate determination in other contexts as well. In zebrafish, loss of Vangl results in misaligned cell division in the dorsal epiblast during gastrulation (Gong et al., 2004), and in the developing mouse brain Vangl2 functions to maintain cortical progenitors *via* ACD by regulating distribution of Pins to orient the mitotic spindle (Lake and Sokol, 2009). Others report that Vangl2 function is critical for post-mitotic re-intercalation of neural keel daughter cells into the neuroepithelium in zebrafish, suggesting that Vangl2 also functions after mitosis to re-establish polarity following cell division (Ciruna et al., 2006). While these studies highlight the conservation of Vangl regulation of both ACD and mitotic spindle orientation, the mechanistic underpinnings of Vangl function in these contexts was not investigated and remains poorly understood.

Vangl is also involved in the maintenance and organization of adult epithelial tissues. Highly proliferative tissues, such as mammalian skin cells, must maintain epithelial organization as they undergo continual rounds of cell division. Devenport et al. (2011) identified mitotic internalization of PCP components as a mechanism to maintain tissue-wide PCP by preventing actively dividing cells from providing aberrant polarity signals to adjacent cells during mitosis. Mechanistically, Vangl2, Fzd6, and Celsr1, which are asymmetrically distributed anterior-posterior during interphase, are selectively internalized at the onset of mitosis in a Celsr1-dependent manner (Devenport et al., 2011). Follow-up studies revealed that while Fzd6 and Celsr1 are internalized from the membrane of dividing cells and adjacent neighbors, internalized Vangl2 is derived from adjacent cells only. Vangl2 at the membrane of actively dividing cells is resistant to internalization, suggesting that Vangl2 is critical for re-establishing both tissue-wide planar polarity and asymmetric complex localization within the daughter cells (Heck and Devenport, 2017). Future studies focused on elucidating the function of retained Vangl2 are required, but it seems likely that retained Vangl2 provides initial polarity cues and serves as a scaffold for the assembly of polarity complexes in the daughter cells.

An emerging role for Vangl in mediating stem cell maintenance and differentiation has been identified in several tissue types. For example, Vangl2 expression contributes to skeletal muscle regeneration by increasing stem cell self-renewal in a nitric oxide-dependent manner (Buono et al., 2012). Similarly, Vangl2 expression in the mammary gland correlates with BMI1, an important regulator of mammary stemness, and Vangl2 may contribute to mammary stem cell maintenance by mediating luminal cell turnover through regulation of proliferation and apoptosis (Smith et al., 2019). Wnt/PCP signaling is also implicated in directing cell-fate specification in intestinal stem cell lineages, but the contribution of Vangl in this process has not yet been explored (Böttcher et al., 2021). Although the exact mechanisms by which Vangl is involved in these processes remains largely undefined, it is possible that Vangl is mediating ACD, a critical mechanism for self-renewal of the stem cell niche (Yamashita et al., 2010), in these contexts.

Very recently, Gong et al. (2021), described a mechanism by which Vangl2 localized to the lysosomal membrane negatively regulates mesenchymal stem cell differentiation in osteoblasts. Here, Vangl2 localized to detergent-resistant microdomains on the lysosome directly binds LAMP2A *via* its PDZ binding motif and targets it for degradation. This results in cathepsin A-mediated degradation of LAMP2A, regulating the activity of lysosomes that have been primed for protein degradation. Interestingly, the authors claim that Vangl functions independently of PCP signaling in this context, suggesting additional roles for Vangl as a scaffold in alternative pathways.

## Vangl as a Key Regulator of Tumor Progression

Given the observation that developmental pathways are often hijacked and reactivated by tumor cells, it is not surprising that core components of PCP signaling have been implicated in tumorigenesis. Indeed, both Vangl1 and Vangl2 are dysregulated in diverse tumor types and, consistent with its role in mediating cell motility events in development, Vangl contributes to cancer cell migration, invasion, and metastasis [reviewed in (Hatakeyama et al., 2014; VanderVorst et al., 2018)]. Vangl has been reported to scaffold both mediators and negative regulators of PCP signaling, suggesting that Vangl interacting partners are probably context- and tissue-dependent. Conflicting subcellular localization patterns for Vangl have been observed in migratory breast cancer cells, indicating that classic PCP asymmetry may not be conserved in this context. One study observed that Vangl1 complexes with the polarity protein Scrib and the adaptor protein NOS1AP at the leading edge of lamellipodia, suggesting that the assembly of Vangl1-Scrib-NOS1AP complexes at the leading-edge of migratory breast cancer cells may promote engagement of downstream signaling components critical to breast cancer cell polarity and motility (Anastas et al., 2012). Alternatively, Luga et al. (2012) observed that Wnt11 mediates the asymmetric localization of the Wnt/PCP components within migratory protrusions of migrating breast cancer cells. Here, Fzd6 and Dvl1 localized to the protrusive fronts of migratory breast cancer cells while Vangl1 was observed to localize to the base of migratory protrusions with Pk1. These authors hypothesized that Vangl serves as a scaffold for its binding partner Pk1 to inhibit the development of non-productive migratory protrusions to maintain directed cell motility. While both studies ultimately implicate Vangl as a critical regulator of breast cancer cell migration, these contradictory findings may stem from differences in systems using ectopically expressed versus endogenous proteins, and the addition or exclusion of Wnt stimulation.

Some studies suggest that Vangl2 is also a scaffold for mediators of breast cancer cell proliferation. For example, Vangl2 binds p62/SQSTM1, an intracellular adaptor protein, in the late endosomal compartment, which engages JNK signaling and drives breast cancer cell migration and anchorage-dependent and -independent growth (Puvirajesinghe et al., 2016). Further, Pk1 has been implicated as an important contributor to breast cancer progression. In breast cancer cells, Pk1 interacts with Mink1 and RICTOR to engage Akt signaling-mediated reorganization of the actin cytoskeleton to promotes breast cancer cell migration (Daulat et al., 2016). Similarly, Pk1 was shown to interact with RhoGAPs Arhgap21/23 to coordinate RhoA activity and drive cytoskeletal rearrangements and cell motility (Zhang et al., 2016). Because Vangl is a known and common binding partner of Pk, it is tempting to speculate that it may be functioning as a scaffold on which Pk1 assembles in these contexts.

While the mechanistic underpinnings of Vangl contribution to tumorigenesis have been best described in breast cancer cells, Vangl has also been implicated in other tumor types. For example, in gastric tumor cells, Vangl1 drives tumor cell invasiveness and metastasis and binds to the C-terminal domain of KAI1 (CD82), a tetraspanin glycoprotein. However, it is unclear whether this interaction is required for Vangl1-mediated tumor cell behaviors in this context (Lee et al., 2004). Vangl1 has also been reported to complex with ErbB4 and phosphorylated Dvl2 through its C-terminal cytoplasmic tail, which engages c-Jun and AP-1 to drive colorectal cancer cell (CRC) invasion and anchorage-dependent growth (Bae et al., 2014). Consistently, Vangl1 appears to function as a scaffold for Dvl and PKCδ. Here, the DEP domain of Dvl binds to the C-terminus of Vangl1, creating a functional Vangl1-Dvl-PKCδ tertiary complex that mediates downstream signaling events through Erk/AP-1 and drives actin cytoskeletal rearrangements to promote CRC invasiveness (Kho et al., 2009).

Vangl also serves as a scaffold for negative regulators of the pathway to drive tumor suppressive outcomes. For example, in melanoma cells, Vangl1 and Vangl2 interact with the E3 ubiquitin ligase RNF43. In this context, Vangl2 colocalizes with RNF43 at the plasma membrane and undergoes RNF43-mediated ubiquitination that results in Vangl2 proteasomal degradation and inhibition of Wnt5a-mediated cell invasion (Radaszkiewicz et al., 2021). We (Wald et al., 2017) and others (Sun et al., 2017) have implicated Vangl as a scaffold for the E3 ubiquitin ligase Nrdp1, and have identified that the coiled-coil domain of Nrdp1 is critical for interaction with Vangl1 and Vangl2 (Wald et al., 2017). In glioblastoma, the C-terminal cytoplasmic tail of Vangl serves as a scaffold for Nrdp1 to mediate K63-polyubiquitination of Dvl, which prevents the recruitment of Dvl to membrane localized Fzd, suppressing downstream signaling and cell motility (Wald et al., 2017). In contrast, loss of Vangl2 in fibrosarcoma cancer cells appears to increase MMP2 and MMP14 resulting in enhanced cellular migration and invasion (Cantrell and Jessen, 2010). Mechanistically, Vangl2 regulation of MMP2 activity is dependent upon the integrin ανβ3, which binds directly to Vangl2 (Jessen and Jessen, 2017). Vangl has also been reported to contribute to tumor progression in many other contexts (Kaucká et al., 2013; Yoon et al., 2013; Lee et al., 2015; Hayes et al., 2018), but Vangl binding partners and mechanistic insight are absent from these studies. Taken together, these findings suggest that Vangl function as a scaffold is conserved across development and tumorigenesis, and that Vangl binding partners are highly tissue- and context- dependent.

## Conclusion

Studies across various model organisms indicate that Vangl contributes to diverse biological functions critical for proper embryonic development and the maintenance of complex tissue structures. The role of Vangl in establishing planar polarity in vertebrates and non-vertebrates has been well-studied and the molecular mechanisms underlying Vangl function in a variety of tissue types have been described. More recently, Vangl has been identified as a key mediator and regulator of cell motility events that are both fundamental to developing embryos and exploited in tumorigenesis. While the molecular insights into Vangl function in these contexts is more limited, we can appreciate the diversity and breadth of signaling molecules with which Vangl engages and interacts (Figure 2). A picture has begun to emerge where Vangl serves as a master scaffolding protein on which both core PCP components and tissue- or context- dependent effectors assemble to execute signaling events with precise spatiotemporal kinetics. To truly understand the molecular mechanisms by which Vangl regulates developmental processes, and to identify potential avenues for intervention in diseases that result from Vangl dysregulation, identification of additional Vangl binding partners is essential.

Despite significant advances in our understanding of Vangl function, many questions remain, and we still have much to uncover. For example, while both Vangl1 and Vangl2 have been implicated in various aspects of development and disease, we generally observe that disruption of Vangl2 produces more severe phenotypes than disruption of Vangl1 (Torban et al., 2008), and we have examples where Vangl1 is dispensable for some aspects of development, such as mammary gland development, whereas Vangl2 is not (Smith et al., 2019). This raises questions concerning the contexts in which Vangl1 and Vangl2 can compensate for each other and contexts in which they have distinct functions. Vangl1 and Vangl2 have nearly identical domain structures and biochemical properties, and in some cases have overlapping binding partners suggesting they could function interchangeably. Some studies suggest that the importance of one Vangl over the other in some biological contexts arises from differences in spatiotemporal expression *in vivo*. For example, during zebrafish gastrulation, Vangl2 is expressed broadly throughout development whereas Vangl1 is expressed later in development and limited to nervous system tissues (Jessen and Solnica-Krezel, 2004). Contribution of Vangl to the establishment of planar polarity and tissue morphogenesis, and the reactivation of Vangl-dependent signaling in cancer have generated many questions regarding mechanisms that remain to be explored.
